# What People “Like”: Analysis of Social Media Strategies Used by Food Industry Brands, Lifestyle Brands, and Health Promotion Organizations on Facebook and Instagram

**DOI:** 10.2196/10227

**Published:** 2018-06-14

**Authors:** Karen Michelle Klassen, Emily S Borleis, Linda Brennan, Mike Reid, Tracy A McCaffrey, Megan SC Lim

**Affiliations:** ^1^ Monash University Notting Hill Australia; ^2^ RMIT University Melbourne Australia; ^3^ Burnet Institute Melbourne Australia

**Keywords:** nutrition, social media, Facebook, Instagram, health promotion

## Abstract

**Background:**

Health campaigns have struggled to gain traction with young adults using social media, even though more than 80% of young adults are using social media at least once per day. Many food industry and lifestyle brands have been successful in achieving high levels of user engagement and promoting their messages; therefore, there may be lessons to be learned by examining the successful strategies commercial brands employ.

**Objective:**

This study aims to identify and quantify social media strategies used by the food industry and lifestyle brands, and health promotion organizations across the social networking sites Facebook and Instagram.

**Methods:**

The six most engaging posts from the 10 most popular food industry and lifestyle brands and six health promotion organizations were included in this study. A coding framework was developed to categorize social media strategies, and engagement metrics were collected. Exploratory linear regression models were used to examine associations between strategies used and interactions on Facebook and Instagram.

**Results:**

Posts from Facebook (143/227, 63.0%) and Instagram (84/227, 37.0%) were included. Photos (64%) and videos (34%) were used to enhance most posts. Different strategies were most effective for Facebook and Instagram. Strategies associated with higher Facebook interactions included links to purchasable items (beta=0.81, 95% CI 0.50 to 1.13, *P*<.001) featuring body image messages compared with food content (beta=1.96, 95% CI 1.29 to 2.64, *P*<.001), and where the content induced positive emotions (beta=0.31, 95% CI 0.04 to 0.57, *P*=.02). Facebook interactions were negatively associated with using pop culture (beta=–0.67, 95% CI –0.99 to –0.34, *P*<.001), storytelling (beta=–0.86, 95% CI –1.29 to –0.43, *P*<.001) or visually appealing graphics (beta=–0.53, 95% CI –0.78 to –0.28, *P*<.001) in their posts compared with other strategies. Posting relatable content was negatively associated with interactions on Facebook (beta=–0.29, 95% CI –0.53 to –0.06, *P*=.01), but positively associated on Instagram (beta=0.50, 95% CI 0.05 to 0.95, *P*=.03). Instagram interactions were negatively associated with weight loss (beta=–1.45, 95% CI –2.69 to –0.21, *P*=.02) and other content (beta=–0.81, 95% CI –1.57 to –.06, *P*=.04) compared with food content.

**Conclusions:**

Health promotion professionals and organizations can improve engagement using positive messaging and tailoring posts appropriate for different social media channels.

## Introduction

### Background

Social media is used almost ubiquitously, especially by young adults, with Facebook being the most common platform [1,2]. More than 80% of Australian young adults are accessing social media platforms at least once per day, particularly during key times of day associated with choosing foods, such as first thing in the morning and at lunch [3]. Since November 2007, Facebook has allowed brands and companies to create profiles [4], and since then, brands have been successful in using the features offered by social media to communicate with the public. Users are not only willingly engaging with brands, but also disseminating brand content to their circle of friends, thereby increasing brand reach [4].

The food industry has been very successful in marketing via social media [5] often winning prizes for marketing innovation [6]. Social media feeds can include both posts from brands individuals have chosen to follow, as well as advertisements. Food industry brands particularly target adolescents and young adults on social media and being exposed to these advertisements appears to influence their attitudes and intentions [7] and may influence their behavior as a result [8]. Young adults have a higher intake of sugar-sweetened beverages and are more likely to consume fast foods compared with other age groups [9]. It is possible that constant exposure to messages from food and beverage brands via social media increases or reinforces these unhealthy eating patterns.

Social media is also being widely used for health promotion [10-12] by health professionals, researchers, government, and non-governmental organizations. Identifying a target audience and tailoring a suitable health message remains important for health promotion design for both traditional and social media campaigns [13]. Benefits of using social media for health promotion are increased reach and interaction [10]; despite this, health promotion campaigns run via social media have traditionally struggled to reach and engage with large numbers of people [14].

In previous research, we found that similar marketing strategies were being used by alcohol brands and health promotion agencies to engage users. Such strategies included posting visually attractive content and linking posts to consumption cultures [14]. Other alcohol and sexual health research also found that consistency of posting and interaction between brands and users are associated with increased success [15,16].

Social media personalities, or “Influencers”, have more recently been identified as being a strategic and powerful avenue for product promotion [17]. Social media Influencers can be defined as individuals or groups of individuals who can shape attitudes and behaviors through online channels [18]. What makes these Influencers so successful appears to be their capacity to engage with users and develop a level of trust [17,19]. According to Nielsen’s 2012 report “Global Trust in Advertising and Brand Messages”, 92% of survey participants stated that they would trust word-of-mouth recommendations above any other form of advertising. Social media Influencers have overtaken traditional celebrities in their ability to influence purchasing behavior, as users find them more credible and relatable [20]. However, traditional celebrity figures still appear to have a strong influence on lifestyle behaviors and some are even seen as “experts” in these areas [21]. Companies are also using Influencers and celebrities to enhance their brand and take inspiration from strategies used by social media Influencers in their own campaign materials [17,22,23].

Research examining social media strategies used in food and nutrition-related communication is currently lacking. No previous studies have compared user engagement on Facebook and Instagram for food brands, lifestyle brands, and health promotion organizations. In identifying the most successful strategies regarding engagement of users with a post, it will be possible to make recommendations for the improvement of nutrition-related health promotion using social media. A glossary of terms has been provided in Multimedia Appendix 1.

The aim of this study was to analyze the content of popular posts made by food brands, lifestyle brands and health promotion organizations using social media (Facebook and Instagram) and to identify strategies associated with engagement.

## Methods

### Design

This study was a retrospective content analysis that used a mixed-methods exploratory design [24] to analyze content from public posts made on Facebook and Instagram by food industry brands, lifestyle brands and health promotion organizations and examined associations between content strategies and engagement.

### Inclusion criteria

Social media pages or profiles (brands have “pages” on Facebook and “profiles” on Instagram; for consistency, herein referred to as “pages”) were selected for inclusion if they posted nutrition or food content and were active at the time of data collection. The top food industry brands, lifestyle brands (defined as any individual or non-food industry brand that creates content on social media that includes food or nutrition information and can include Influencers who have created a brand) and health promotion organizations in Australia were determined by the number of Australian fans (when a user follows a page, the user becomes a fan of the page) on Facebook. Brands could be global or Australian as long as they had a social media presence in Australia. As this was an exploratory study, 10 lifestyle and food industry brands and only six health promotion organizations were included due to the small number of organizations running nutrition-related health promotion campaigns on social media. This number was chosen to provide sufficient data to undertake exploratory analyses. The top 10 food industry and top 10 lifestyle brands were identified through Socialbakers [25]. For food industry brands, the following filters were selected: Facebook, brands/Fast-moving consumer goods (FMCG) Food/All FMCG Food, Australia (total fans). For lifestyle brands, the filters included: Facebook, celebrities, Australia (total fans); Facebook, entertainment, online show, Australia (total fans); and Facebook, community, lifestyle, Australia (total fans).

The top health promotion organizations (n=6) were identified using the filters: Facebook, Society/All Society, Australia (total fans). Additionally, an online search of Australian health promotion organizations was conducted, and all partners of the Communicating Health project and organizations were considered for inclusion if they ran a nutrition and health-related campaign on social media in the past five years.

### Data Collection

Data were collected for lifestyle and food industry brands from Facebook and Instagram between August and September 2017. Socialbakers Suite [25] monitors and collates data on the activity of millions of brands on social media and reports statistics by country and brand. Socialbakers provided the top six most engaging posts for both Facebook and Instagram during a 30-day period; therefore, these top six posts for each platform were used for all included pages. Some brands did not have an Australian Instagram page; therefore, the number of Instagram posts evaluated was less than on Facebook. If less than six posts were available during the 30-day period, data from all available posts were included.

Data were collected for health promotion organizations from Facebook and Instagram by searching Facebook and Instagram using Google Chrome web browser through searching for the selected campaign hashtag; sorting posts by “top posts” and “organization name” and choosing the top six posts. The most well-known and recent campaign for each health promotion organization was identified through online and literature searches.

#### Coding framework

A coding framework identified strategies used in posts by examining the qualitative data collected from the post content (including text, videos, and photographs). The framework was constructed by combining both deductive and inductive strategies (Multimedia Appendix 2). The deductive category development was based on prior research [14], everyday knowledge and logic. The inductive category development was informed by the content of the included posts themselves using open coding, a technique from grounded theory [26].

The coding process was iterative and continued to develop with previous posts being revisited throughout. Coding was done by three researchers: (1) KK, a nutrition professional and research fellow, (2) EB a nutrition science undergraduate student, and (3) ACY, a medical undergraduate student). Any differences in coding between researchers were discussed and a final decision was made by KK.

#### Engagement metrics

Quantitative data collected included social media engagement. Facebook engagement was measured in the following way: (1) reactions: when a user expresses their reaction to a post by clicking either “like,” “love,” “haha,” “wow,” “sad,” or “angry;” (2) comments: when a user leaves a comment or replies to the post; and (3) shares: when a user “shares” (also referred to as tagging) or reposts the post [27-29]. Interactions are the sum of the number of comments, shares and all reactions. Instagram engagement was measured as follows: (1) likes: when a user clicks “like”, which suggests that a post has resonated with a user in some way [30], and (2) comments. Total Facebook interactions per post were calculated by summing of the number of comments, shares and all reactions (like, love, haha, wow, sad, and angry). Total Instagram interactions per post were calculated by summing the number of comments and likes.

#### Statistical Analysis

Categories developed from the coding framework were transformed into quantitative categorical data. Descriptive statistics provide characteristics for each organization and coding categories. Each coding category was evaluated based on frequency within and between the categories, and similar categories were combined for analysis.

Statistical models constructed were exploratory and inductive due to the paucity of similar research in this area; therefore, a backward, stepwise approach was used. Engagement metric data were positively skewed, therefore log-transformed for analysis. Univariable linear regression models were constructed to determine the variables to be included in the final multivariable regression models. Variables with a *P* value of <.200 in the univariable linear regression models were considered for inclusion in the multivariable models. All categories identified in the coding framework were considered for inclusion in the models.

Multivariable linear regression models were constructed to explore associations of the post content analysis with the engagement measures (ie, the dependent variables): Facebook and Instagram interactions. Models were tested for heteroscedasticity and normality of residuals, and extreme outliers were removed from the models.

Statistical analyses were conducted using Stata (Version 12, College Station, TX, USA).

### Ethics

Ethics approval was received by Monash University Human Research Ethics committee (project 11945). Data presented are anonymized to protect the identity of brands or organizations included in the study.

## Results

A total of 227 posts from health promotion organizations (34/227, 15.0%), food industry brands (79/227, 34.8%) and lifestyle brands (114/227, 50.2%) were analyzed from Facebook and Instagram. Regarding engagement metrics, included health promotion organizations had fewer fans on both Facebook and Instagram than food industry brands and lifestyle brands (Table 1). Health promotion organizations had less of a presence on Instagram, with fewer fans than on Facebook (Table 1).

**Table 1:** Brand engagement metrics.

Engagement metric	Health promotion organizations	Food industry brands	Lifestyle brands
Facebook pages included, n	6	10	10
Instagram profiles included, n	0	7	10
Facebook fans, median (25^th^; 75^th^percentiles)	21,784 (9,896; 56,939)	1,033,517 (804,210; 32,039,808)	1,590,354 (1,142,469; 10,625,219)
Facebook fans in Australia, median (25^th^; 75^th^percentiles)	20,119 (9,896; 51,155)	807,185 (647,250; 908,238)	365,111 (128,804; 531,601)
Facebook page likes, median (25^th^; 75^th^percentiles)	22,034 (9,926; 59,309)	12,999,467 (829,335; 32,046,105)	1,622,860 (1,152,053; 10,912,143)
Instagram fans, median (25^th^; 75^th^percentiles)	4,842 (2,732; 7,836)^a^	87,917 (36,001; 146,825)	328,509 (25,453; 2,140,075)
Facebook posts included in analysis, n	34	51	58
Instagram posts included in analysis, n	0	28	56
Total posts included in analysis, n	34	79	114
Interactions per Facebook post, median (25^th^; 75^th^percentiles)	41 (30; 96)	2,484 (377; 6,219)	3,766 (1,205; 33,825)
Interactions per Instagram post, median (25^th^; 75^th^percentiles)	—^b^	493 (267; 1,417)	8,530 (115; 53, 708)

^a^Data available for n=4 organizations only.

^b^Data not available.

Lifestyle brands had higher engagement with posts on both Facebook and Instagram when compared with both food industry and health promotion organizations (Table 1). Posts on Facebook had more engagement with a median (25^th^; 75^th^percentiles) of 1,763 interactions (165; 7,374) than those on Instagram with 1,582 interactions (211; 18,414).

The proportion of posts using different engagement strategies is shown in Multimedia Appendix 3. For all categories except relationship building and format, the strategies used were diverse across the different organizations. Most posts used photographs (145/227, 64%), with only a few using only text (5/227, 2%). Health promotion organizations used more ‘prompting engagement’ strategies, links to health information, featured fruits, vegetables, and grains, had a more serious tone, used hashtags, had more real-world tie-ins than the other organizations and were the only organization type to present statistics or facts in their posts. Lifestyle brands and health promotion organizations induced more positive emotions than food industry; food industry and lifestyle brands had more links to purchasable items; food industry had the highest product promotion and did not feature people in most of their posts; lifestyle brands were the only group to talk about body image and weight loss and had the most interactions per 1000 fans for both Facebook and Instagram.

Two multivariable regression models were constructed to explore the associations between social media strategies and engagement (measured by interactions) on Facebook and Instagram (Table 2). Facebook interactions were positively associated with lifestyle brands compared with health promotion organizations, including links to purchasable items (beta=0.81, 95% CI 0.50 to 1.13, *P*<.001), featuring body image messages compared with food content (beta=1.96, 95% CI 1.29 to 2.64, *P*<.001), posting videos compared with photos (beta=0.33, 95%CI 0.11 to 0.54, *P*=.004) and where the content induced positive emotions (beta=0.31, 95% CI 0.04 to 0.57, *P*=.02).

Facebook interactions were negatively associated with using pop culture (beta=–0.67, 95% CI -0.99 to –0.34, *P*<.001), story-telling (beta=–0.86, 95% CI –1.29 to –0.43, *P*<.001) or visually appealing graphics (beta=–0.53, 95% CI –0.78 to –0.28, *P*<.001) in their posts compared with other strategies, featuring weight loss compared with food content (beta=–1.06, 95% CI –1.76 to –0.37, *P*=.003), featuring people (beta=–0.42, 95% CI –0.71 to –0.13, *P*=.005), including links to health information (beta=–0.47, 95% CI –0.83 to –0.10, *P*=.01), posting relatable content (beta=–0.29, 95% CI –0.53 to –0.06, *P*=.01) and paying to promote posts (beta=–0.30, 95% CI –0.56 to –0.04, *P*=.03).

Instagram interactions were positively associated with including links to purchasable items (beta=1.32, 95% CI 0.77 to 1.88, *P*<.001) and posting relatable content (beta=0.50, 95% CI 0.05 to 0.95, *P*=.03).

Instagram interactions were negatively associated with weight loss (beta=–1.45, 95% CI –2.69 to –0.21, *P*=.02) and other content (beta=–0.81, 95% CI –1.57 to –0.06, *P*=.04) compared with food content, and with using hashtags.

**Table 2:** Multivariable Linear Regression Models of Facebook and Instagram interactions.

Variables in model		Facebook interactions, log (10)		Instagram interactions, log (10)	
		Standardized beta (95% CI)	*P* value	Standardized beta (95% CI)	*P* value
**Organization type**					
	Health promotion organization	Ref^a^	N/A^b^	N/A	N/A
	Food industry	0.45 (–0.15 to 1.05)	.14	Ref	N/A
	Lifestyle brands	1.42 (0.96 to 1.88)	<.001	0.30 (–0.14 to 0.75)	.18
**Strategies used**					
	Other strategies	Ref	N/A	N/A	N/A
	Pop culture	–0.67 (–0.99 to –0.34)	<.001	N/A	N/A
	Story-telling	–0.86 (–1.29 to –0.43)	<.001	N/A	N/A
	Visually appealing	–0.53 (–0.78 to –0.28)	<.001	N/A	N/A
	Links to purchasable items	0.81 (0.50 to 1.13)	<.001	1.32 (0.77 to 1.88)	<.001
**Post content**					
	Food content	Ref	N/A	Ref	N/A
	Body image content	1.96 (1.29 to 2.64)	<.001	–0.30 (–1.21 to 0.62)	.52
	Weight loss content	–1.06 (–1.76 to –0.37)	.003	–1.45 (–2.69 to –0.21)	.02
	Other content	–0.15 (–0.46 to 0.16)	.34	–0.81 (–1.57 to –0.06)	.04
	Posts that featured people	–0.42 (–0.71 to –0.13)	.005	N/A	N/A
	Links to health information	–0.47 (–0.83 to –0.10)	.01	0.29 (–0.28 to 0.87)	.31
**Post format**					
	Photo	Ref	N/A	N/A	N/A
	Video	0.33 (0.11 to 0.54)	.004	N/A	N/A
	Text	–0.04 (–0.57 to 0.49)	.88	N/A	N/A
	Relatable content	–0.29 (-0.53 to -0.06)	.01	0.50 (0.05 to 0.95)	.03
	Positive emotion	0.31 (0.04 to 0.57)	.02	0.47 (–0.10 to 1.04)	.11
	Promoted post	–0.30 (–0.56 to –0.04)	.03	N/A	N/A
	Uses hashtags	–0.25 (–0.50 to 0.00)	.05	–0.55 (–0.91 to –0.19)	.003
	Optimistic tone of post	–0.22 (–0.49 to 0.05)	.11	N/A	N/A
Number of posts included in model		141	N/A	84	N/A
Adjusted R^2^		78.2%	N/A	60.2%	N/A

^a^Ref: reference category for multivariable linear regression.

^b^N/A: not applicable.

## Discussion

### Principal Findings

This is the first study to identify and quantify the social media strategies utilized by food industry brands, lifestyle brands and health promotion organizations across Facebook and Instagram, to our knowledge. Each organization type used different social media strategies to engage users. The food industry brands attempted to induce appetite and encourage users to eat; health promotion organizations frequently provided statistics and facts and used a more serious tone, while lifestyle brands were positive and relatable. Health promotion organizations were not chosen based on the 10 most popular organizations but were limited to those organizations with lifestyle-related campaigns. Therefore, while health promotion organizations had substantially fewer followers and post interactions, direct comparisons between the number of fans of health promotion organizations and other brands are not applicable.

Links to purchasable items were used by both food and lifestyle brands and were consistently associated with more interactions on both Facebook and Instagram. This is not surprising considering consumers who are fans of such pages are often seeking new products or versions or products and may be engaged in online purchasing or exploration of available offers. Schultz et al [31] found that promotional posts were negatively associated with Facebook post likes, but positively associated with Facebook shares. However, we did not do separate analyses for each interaction type. Many people hate being exposed to advertisements and try to block it from their social media feeds [32], while others do not notice the advertisements to which they are exposed [33]. Food industry brands included in this study had the highest number of Facebook fans but the lowest level of engagement per post per 1000 fans compared with other organization types. Although fewer Australians claim to be following brand pages on social media than in previous years, those who were following brands said they were doing so to receive discounts (54%) and to receive free items acquired from giveaways (48%) [3]. Of the food industry posts analyzed in the current study, 35% were advertising discounts or giveaways, which is similar to other findings [4]. These results suggest that users are engaging with food industry brands for their own gain, financial or otherwise.

Positive emotion-inducing strategies were associated with more interactions on Facebook and Instagram and using an optimistic tone was associated with more interactions on Facebook. Emotion plays a role in the attention and attraction the user experiences towards a post [34]. Participants who experience positive emotions when viewing a post on social media are far more likely to engage with that post than those who do not experience positive emotions [35].

Models for the two social media channels explored, Facebook and Instagram, included different strategies that were statistically significant. For example, posts classified as “relatable” were negatively associated with Facebook interactions but positively associated with Instagram interactions. Posts were classified as “relatable” if they encouraged feelings of friendship between the poster and fan or if the post contained content that is "relatable" to the user. Examples of this included providing practical advice that would apply to their audience or talking about issues they think are important or interesting to their fans (eg, debating the pros and cons of Hawaiian pizza, or what to eat for breakfast). Each social media channel has different features, is used differently by users and therefore strategies should be tailored for each channel [36]. Since its development in 2010, Instagram has become one of the most popular photo sharing applications worldwide [37] and particularly since the introduction of Instagram Stories and Instagram Live allows for immediate engagement with users. Instagram facilitates para social interactions, where imaginary social relationships and interpersonal interactions between the lifestyle personality and the social media user occur [38]. These relationships and interactions can be developed by using some of the strategies observed in the current study: relatable content, use of personal stories, and positive emotion and tone. This form of interaction may help to explain high levels of engagement seen on such posts; users are developing connections to the personality and may treat them similarly, to how they would treat a friend on social media.

Other differences between strategies associated with the different social media channels examined included post format (video versus photographs) and including body image content. Videos and body image content were statistically significantly positively associated with Facebook interactions, but not associated with Instagram interactions. These results emphasize the importance of tailoring messages to suit both the social media platform used and the desired outcome.

### How Can Health Promotion Organizations Enhance Their Social Media Strategies?

Health promotion organizations had less of an overall presence on Instagram. Heldman et al [39] discuss how despite many organizations developing a social media presence, they often lack in the more social aspects. Rather than adapting to this online form of communication, organizations appear to be continuing with many of the strategies used in traditional health promotion [40]. What is evident from our analyses is that different organization types used different strategies in their posts. The use of facts/statistics and less frequent use of an optimistic tone and using real-world tie-ins by health promotion organizations were the biggest differences between the groups. The health promotion organizations analyzed in the current study developed posts that were more serious in tone and often relied on statistics and facts to communicate their intended message. The information provided in the posts was important and frequently linked to further information; however, these posts had minimal engagement from fans. Taken together the engagement metrics and strategies presented in Table 1 and Multimedia Appendix 3 illustrate how building relationships with user/fans/followers are advantageous for lifestyle brands. As health promotion organizations, we should be cognizant of this approach to engage our target audience.

In the past, many health promotion organizations have developed campaigns employing fear as a way to induce behavior change [41]. For some aspects of health promotion (eg, anti-smoking campaigns) there may be room for both approaches, but it remains to be elucidated whether or not this is suitable in organizations communicating food and nutrition messages as this strategy was not used by other organizations in this study.

The coding framework developed in this study can be used as a guide by health organizations who are planning social media campaigns to target young adults.

### Limitations

Limitations of this study included the short time frame of data collected. Evaluating a longer period could identify seasonal differences and improve strategies for creating messages for holidays or special events, and we plan to continue to develop the coding framework and monitor future posts to address this limitation. We included posts that did not contain food or health-related information which limited our ability to examine strategies that are particularly effective for food-related messages. We chose to include posts that were not directly about food or nutrition as long as they were from a brand (or profile) that posted about food or nutrition. The diversity of content from these brands, particularly the lifestyle brands demonstrates the importance of relationship building and indicates that people are getting food and nutrition-related information from pages posting diverse content. Furthermore, although interactions provide good measures of user engagement, the level to which users take on and use this information cannot be determined from these metrics alone. Although traditional social media engagement statistics (reactions, comments, shares, and interactions) indicate the number of users that interact with social media posts, they do not indicate any resulting behavior change nor those who view, process and interact with the content “offline” (eg, the lurkers [42]), nor do they indicate behavior change as a result of interacting with the posts [7,43]. Further research could analyze the content of the comments on posts to gauge the quality of interaction between the page owner and follower and to gain insight into users’ intention to behave.

### Conclusions

This unique, exploratory study examined "real-life" social media posts with a sample size sufficient to create a coding framework and to create exploratory models.

Social media content should be tailored to suit not only the target audience but also the social media channel being used and the desired engagement. Health promotion practitioners and organizations can learn from other types of brands and consider using few statistics and more positive content to relay healthy eating messages.

## Appendix

Multimedia Appendix 1 Glossary of terms.

Multimedia Appendix 2 Coding framework.

Multimedia Appendix 3 Coding framework characteristics or strategies used for social media posts.

## Abbreviations

AFG: Australian Food Guide
AGtHE: Australian Guide to Healthy Eating
FMCG: Fast-moving consumer goods

## References

[1] Greenwood S, Perrin A, Duggan M (2016). Social Media Update.

[2] Sensis (2016). Sensis Social Media Report.

[3] Sensis (2017). Sensis Social Media Report.

[4] Freeman Becky, Kelly Bridget, Baur Louise, Chapman Kathy, Chapman Simon, Gill Tim, King Lesley (2014). Digital junk: food and beverage marketing on Facebook. Am J Public Health.

[5] Freeman B, Kelly B, Vandevijvere S, Baur L (2016). Young adults: beloved by food and drink marketers and forgotten by public health?. Health Promot Int.

[6] Colchester J, Hargreves T WARC Trends.

[7] Buchanan L, Kelly B, Yeatman H (2017). Exposure to digital marketing enhances young adults' interest in energy drinks: An exploratory investigation. PLoS One.

[8] Fishbein M (2000). The role of theory in HIV prevention. AIDS Care.

[9] Australian Bureau of Statistics (2015). Australian health survey: First results Australia 2014-2015.

[10] Moorhead SA, Hazlett DE, Harrison L, Carroll JK, Irwin A, Hoving C (2013). A new dimension of health care: systematic review of the uses, benefits, and limitations of social media for health communication. J Med Internet Res.

[11] Levac J, O'Sullivan T (2010). Social Media and its Use in Health Promotion. Revue interdisciplinaire des sciences de la santé - Interdisciplinary Journal of Health Sciences.

[12] Balatsoukas P, Kennedy CM, Buchan I, Powell J, Ainsworth J (2015). The Role of Social Network Technologies in Online Health Promotion: A Narrative Review of Theoretical and Empirical Factors Influencing Intervention Effectiveness. J Med Internet Res.

[13] Korda H, Itani Z (2013). Harnessing social media for health promotion and behavior change. Health Promot Pract.

[14] Lim MS, Hare JD, Carrotte ER, Dietze PM (2016). An investigation of strategies used in alcohol brand marketing and alcohol-related health promotion on Facebook. DIGITAL HEALTH.

[15] Burton S, Dadich A, Soboleva A (2013). Competing Voices: Marketing and Counter-Marketing Alcohol on Twitter. Journal of Nonprofit & Public Sector Marketing.

[16] Veale HJ, Sacks-Davis R, Weaver ER, Pedrana AE, Stoové MA, Hellard ME (2015). The use of social networking platforms for sexual health promotion: identifying key strategies for successful user engagement. BMC Public Health.

[17] Uzunoğlu E, Misci Kip S (2014). Brand communication through digital influencers: Leveraging blogger engagement. International Journal of Information Management.

[18] Freberg Karen, Graham Kristin, McGaughey Karen, Freberg Laura (2011). Who are the social media influencers? A study of public perceptions of personality. Public Relations Review.

[19] Liu Shixi, Jiang Cuiqing, Lin Zhangxi, Ding Yong, Duan Rui, Xu Zhicai (2015). Identifying effective influencers based on trust for electronic word-of-mouth marketing: A domain-aware approach. Information Sciences.

[20] Djafarova E, Rushworth C (2017). Exploring the credibility of online celebrities' Instagram profiles in influencing the purchase decisions of young female users. Computers in Human Behavior.

[21] Hoffman SJ, Tan C (2015). Biological, psychological and social processes that explain celebrities' influence on patients' health-related behaviors. Arch Public Health.

[22] Chapman S (2012). Does celebrity involvement in public health campaigns deliver long term benefit? Yes. BMJ.

[23] Rayner Geof (2012). Does celebrity involvement in public health campaigns deliver long term benefit? No. BMJ.

[24] Creswell J (2003). Research design: Qualitative, quantitative and mixed methods approaches.

[25] Socialbakers Statistics.

[26] Glaser BG, Strauss AL (1967). The discovery of grounded theory: Strategies for qualitative research.

[27] Facebook Brand Resource Centre Reactions.

[28] Simply Measured Facebook Comment.

[29] Simply Measured Facebook Share.

[30] Simply Measured Instagram Like.

[31] Schultz CD (2017). Proposing to your fans: Which brand post characteristics drive consumer engagement activities on social media brand pages?. Electronic Commerce Research and Applications.

[32] Crozier M (2016). Engaging young people online.

[33] Media Dynamics Incorporated (2014). Adults Spend Almost 10 Hours Per Day With The Media, But Note Only 150 Ads.

[34] Stieglitz S, Dang-Xuan L (2013). Emotions and Information Diffusion in Social Media—Sentiment of Microblogs and Sharing Behavior. Journal of Management Information Systems.

[35] Smith S (2013). Conceptualising and Evaluating Experiences with Brands on Facebook. International Journal of Market Research.

[36] Coelho RLF, Oliveira DSD, Almeida MISD (2016). Does social media matter for post typology? Impact of post content on Facebook and Instagram metrics. Online Information Review.

[37] Lee E, Lee J, Moon JH, Sung Y (2015). Pictures Speak Louder than Words: Motivations for Using Instagram. Cyberpsychol Behav Soc Netw.

[38] Ward J (2016). A Content Analysis of Celebrity Instagram Posts and Parasocial Interaction. Elon Journal of Undergraduate Research in Communications.

[39] Heldman AB, Schindelar J, Weaver JB (2013). Social Media Engagement and Public Health Communication: Implications for Public Health Organizations Being Truly “Social”. Public Health Rev.

[40] Neiger BL, Thackeray R, Van Wagensen SA, Hanson CL, West JH, Barnes MD, Fagen MC (2012). Use of social media in health promotion: purposes, key performance indicators, and evaluation metrics. Health Promot Pract.

[41] Soames JRF (1988). Effective and ineffective use of fear in health promotion campaigns. Am J Public Health.

[42] Merchant Gina, Weibel Nadir, Patrick Kevin, Fowler James H, Norman Greg J, Gupta Anjali, Servetas Christina, Calfas Karen, Raste Ketaki, Pina Laura, Donohue Mike, Griswold William G, Marshall Simon (2014). Click “like” to change your behavior: a mixed methods study of college students' exposure to and engagement with Facebook content designed for weight loss. J Med Internet Res.

[43] Freeman B, Potente S, Rock V, McIver J (2015). Social media campaigns that make a difference: what can public health learn from the corporate sector and other social change marketers?. Public Health Res Pract.

