# Supplementary material for: What People “Like”: Analysis of Social Media Strategies Used by Food Industry Brands, Lifestyle Brands, and Health Promotion Organizations on Facebook and Instagram
Source: J Med Internet Res. 2018 Jun 14;20(6):e10227. doi: 10.2196/10227 (PMC6024098; doi:10.2196/10227)
Supplement: Multimedia Appendix 1 [file jmir_v20i6e10227_app1.pdf]

Multimedia Appendix 1. Glossary of terms

Term	Definition	Adapted from reference and/or example
Brand	What the public think, both factually and emotionally, when they hear a brand name. It can be a design, sign, symbol, words or a combination of these employed in creating a reputation that identifies a product.	[1]
Facebook or Instagram comment	When a user leaves a comment or replies to the post.	[2]
Facebook fan	When a user follows a page, the user becomes a "fan" of the page on Facebook.	[3]
Facebook interactions	Facebook interactions are the sum of the number of comments, shares and all reactions.	
Facebook page	A business account on Facebook to promote business materials. Brands have "pages" on Facebook.	[4, 5] Celebrity chef Pete Evans has a Facebook page "@paleochefpeteevans" and a personal Facebook profile "pete.evans.5473"
Facebook profile	Individuals have "profiles" on Facebook i.e. a personal account where you communicate on a personal level.	See "Facebook page" for an example.
Facebook reactions	When a user expresses their reaction to a post by clicking either "like", "love", "haha", "wow", "sad", or "angry".	[2, 6]
Facebook share	When a user "shares" (also referred to as tagging) or reposts the post.	[7]
Health promotion organization	An organization that aims to promote health by "enabling people to increase control over the determinants of health and thereby improve their health" using a variety of strategies, including health education, mass media, community development and community engagement processes.	[8, 9] VicHealth and the American Heart Association are examples of health promotion organizations
Instagram interactions	Instagram interactions are the sum of the number of likes and number of comments.	

Instagram likes	When a user clicks "like", which suggests that a post has resonated with a user in some way	[10]
Instagram profile	An Instagram profile shows your bio and Instagram posts. Brands have "pages" on Facebook and "profiles" on Instagram, but for consistency, this manuscript refers to both as "pages".	[11]
Lifestyle or Social Media Influencer	Social media Influencers can be defined as individuals or groups of individuals who have the ability to shape attitudes and behaviors through online channels	[12, 13] Kayla Itsines [14]
Lurkers also "lurking"	Individuals on social media who view information, but do not necessarily comment or share	[15]
Post	A Facebook status update or an item (such as a photo or video) on a social media channel, blog or forum.	[16]
Social media engagement	Social media engagement refers to the acts of talking to, messaging or otherwise interacting with other people on social networks. It encompasses many different types of actions on social media (see Facebook and Instagram interactions). Simply put, social media engagement is any interaction users have with other users on social media.	[16]
Social media "page"	This term is used throughout this manuscript to denote a Facebook or Instagram page or profile.	

References:

1. McLaughlin J. 2011. What is a Brand, Anyway? https://www.forbes.com/sites/jerrymclaughlin/2011/12/21/what-is-a-brand-anyway/#4d92fe142a1b. Archived at: http://www.webcitation.org/6wZhOVh9K
2. Simply Measured. 2017. Facebook Comment. http://simplymeasured.com/definition/facebook-comment/#sm.0000k04rm7dpsdfbw9c1ec85xha7f. Archived at: http://www.webcitation.org/6wZezldXF
3. Technopedia Inc. 2018. Facebook Fan. https://www.techopedia.com/definition/15436/facebook-fan. Archived at: http://www.webcitation.org/6xVTd3yQN
4. Ramos J. 2014. Facebook Page vs Facebook Profile: Do You Know The Difference? https://blog.hootsuite.com/facebook-page-vs-facebook-profile/. Archived at: http://www.webcitation.org/6xVTeer6H
5. Simply Measured. 2017. Facebook Page. https://simplymeasured.com/definition/facebook-page/#sm.00190gp1n10yzdo5t731u6llk3j2s. Archived at: http://www.webcitation.org/6wZetskGd
6. Facebook Brand Resource Centre. 2016. Reactions. https://en.facebookbrand.com/assets/reactions. Archived at: http://www.webcitation.org/6wZecN86W
7. Simply Measured. 2017. Facebook Share. http://simplymeasured.com/definition/facebook-share/#sm.0000k04rm7dpsdfbw9c1ec85xha7f. Archived at: http://www.webcitation.org/6wZevjpKb

8. Australian Health Promotion Association. What is health promotion? https://www.healthpromotion.org.au/our-profession/what-is-health-promotion. Archived at: http://www.webcitation.org/6xVTgvdlR
9. World Health Organization. 2018. Health promotion. http://www.who.int/topics/health_promotion/en/.
10. Simply Measured. 2017. Instagram Like. https://simplymeasured.com/definition/instagram-like/#sm.0000k04rm7dpsdfbw9c1ec85xha7f. Archived at: http://www.webcitation.org/6xVTSmZTM
11. Instagram. Profile. https://help.instagram.com/438970006178813.
12. Mediakix Team. 2017. What is an Instagram Influencer? Definition, examples, & insights. http://mediakix.com/2017/07/instagram-influencer-definition-examples/#gs.by0IpSg. Archived at: http://www.webcitation.org/6xVTjGQtL
13. Freberg K, Graham K, McGaughey K, Freberg LA. Who are the social media influencers? A study of public perceptions of personality. Public Relations Review. 2011 2011/03/01/;37(1):90-2. doi: https://doi.org/10.1016/j.pubrev.2010.11.001.
14. Itsines K. 2018. kayla_itsines. https://www.instagram.com/kayla_itsines/. Archived at: http://www.webcitation.org/6xVTkD28i
15. Technopedia Inc. 2018. Lurker. https://www.techopedia.com/definition/8155/lurker. Archived at: http://www.webcitation.org/6xVTlv3Ev
16. Fontein D. 2016. The Social Media Glossary: 226 Essential Definitions. https://blog.hootsuite.com/social-media-glossary-definitions/#P. Archived at: http://www.webcitation.org/6xVTn4Sbk
