# Supplementary material for: What People “Like”: Analysis of Social Media Strategies Used by Food Industry Brands, Lifestyle Brands, and Health Promotion Organizations on Facebook and Instagram
Source: J Med Internet Res. 2018 Jun 14;20(6):e10227. doi: 10.2196/10227 (PMC6024098; doi:10.2196/10227)
Supplement: Multimedia Appendix 2 [file jmir_v20i6e10227_app2.pdf]

Multimedia Appendix 2. Coding framework.

Classification	Social media strategy	Description and method of collection	Examples[a]
**Format**			
	Photo	Photo with text, photo only, shared photo with text, shared photo no text	NA[b]
	Video	Video with text, video only, shared video with text, shared video no text, Gif only, gif with text	NA
	Text	Text only, text with preview link	NA
**Content topic**			
	Food/beverage	Comments on or refers to food and/or beverages	NA
	Body image	Comments on or refers to body image or is promoting a specific body image, includes picture of pregnant body	NA
	Physical activity	Shows or promotes user to participate in physical activity	NA
	Weight loss	Shows or promotes losing weight	NA
	Other	Comment on or refers to another topic	NA
**Prompting engagement**			
	Question	Poll (prompts users to vote for an option), Q&A (asks users to send in questions to be answered), question (asks a question, quiz, etc.)	**“**Getting bored of your breakfast granola?“
	Action	Challenge (prompts users to accept/deny a challenge), tagging (prompts users to tag others in post), sign-up (for further participation, event, competition, etc.), game (prompts activities), fill-in the blank (prompts users to complete a sentence/paragraph), competition (prompts users to complete a set of instructions for a chance to win)	“Girls Tag a friend who needs this safe for their snacks! 👌👌😂😂”
**Relationship-building**			
	Reply	**E**ither directly or as a general reply to users	“Join me for 'live' Q & A - jump on and say Hi! 🙏🙏”

	Other strategies	Posting user-generated content (posts either reposted or obtained from users and shared/posted giving credit to original poster), event photos (from an event held by the poster) or other organization content (originally from another organization)	“xxx is running a free ‘girls only’ surfing clinic with Manly Surf School. Register your interest below by commenting and sharing.”
	Relatable content[c]	Post content that is relatable to user; build friendships (encourages feelings of friendship towards poster); motivate and encourage users to engage in an action	"DON'T WATCH ME, you're making me nervous" tag a friend who can't park. 🤦🏼‍♀️😂😂🚙🚙💥” OR<br>“You HAVE to make this giant cinnamon roll to share with your friends 😱😱!”
**Emotion-inducing[d]**			
	Appetite	Evokes emotions towards food	NA
	Positive	Evokes feeling of attraction (non food-related appealing, evoked desire), awe-inspiring (impressed or amazed), aww (response to something cute) [27], heartfelt (deeply sincere), humour, inspirational (encouraging or motivational)	*“Ummmm how adorable is my little man 😍 cannot even deal!!!!!!!!!! #Allmine ❤ mummy and daddy so proud ! .”*
	Negative	Evokes fear or outrage (offence or insult)	NA
**Association with success**			
	Physical association with success	Health, sporting, weight loss	“These results are just amazing. At day 35, xxx had lost 10 kgs.”
	Social	Obtaining friends (or sexual success) from the product or information promoted in the post	NA
**Real-world tie-ins**			
	Real-world tie-ins	Links to events (users may attend), culture (music, TV, travel, holiday), celebrities, experts, locations (check-ins), sponsorships (partnerships), past events	“Great to have xxx [football club] backing our #H30Challenge! Can you join them in giving up sugary drinks and making the switch to water for 30 days?”
**Encouragement to eat**			

	Recipe provided	NA	“CABBAGE AND BACON SOUP, from our cookbook ‘Xxxx. Tag, share and enjoy!”
	Food shown	NA	NA
	Encouragement to make balanced choices and drink water	NA	NA
	Occasion specific	Eat or drink at a specific time (breakfast, lunch or dinner; study time), day (a specific event or competition), or place (a specific restaurant, event or location)	NA
**Encouragement to exercise**			
	Encouragement to exercise	Shows someone exercising, or describes results of exercise; talks about exercising; describes a workout; occasion-specific (exercise at a specific time, day or place); uses statistics to motivate exercise	“Warm ups and cool downs are an essential part of training.💪 Make sure you stretch all major muscle groups before and after workouts to avoid injuries💪”
**Link to purchasable/downloadable item(s)**			
	Link to purchasable/ downloadable item(s)	Includes links to products, apps, downloads, implied; indirect links can include links to purchasable/downloadable items on the brands’ profile page	NA
**Links to health information**			
	Links to health information	Includes direct or indirect links to information about health (can include campaign material, personal health stories, government websites, etc.)	NA
**Tone of post**			
	More serious	Informative, serious, thoughtful	NA
	Positive	Excited, joyful	NA
	Optimistic[d]	Humorous	“Obviously I am beyond happy to be having a baby, but I wanted to talk about some of the things that helped

			me become "happy" before getting pregnant:"
	Negative[e]	Pessimistic, sad	NA
**Product promotion**			
	Product promotion	Advertises a purchasable item	NA
**Strategies used**			
	Fast paced	Snappy or short videos	NA
	Promotion	Free item, giveaway, price promotion (advertises a discounted or limited time offer)	NA
	Pun	Uses words in a humorous way, includes innuendos and rhymes	NA
	Life hack	Post suggests way(s) for user to make daily activities easier/more effective (eg, quick cooking tips)	NA
	Pop culture	References popular culture, uses memes	"Hunger is coming… Which House of Xxxxeros are you backing for the Golden Throne?"
	Statistics/facts	Provides statistics or facts to inform users	"Did you know sports drinks contain up to 13 tsp of sugar?"
	Story	A testimonial or personal experience told	NA
	Visually appealing	Nice to look at; uses innovative photography, design or video methods	NA
	Quote	Text or a picture of text taken from another source	NA
**Diet assessment of food**			
	Using the Australian Dietary Guidelines food groups [28]	The most dominant food group featured in the post:<br>Discretionary<br>Dairy or protein foods<br>Fruits and Vegetables<br>Grains	NA

[a]Quotes from posts included to provide examples of the categories. Some categories were determined from the photographs or videos; therefore, examples are not provided.

[b]NA: not available.

[c]Relatable and optimistic content were included as separate categories in statistical analysis.

[d]Often emotions are induced through photographs and videos, therefore difficult to find textual examples.
[e]No negative examples were found in this analysis.
