# Supplementary material for: What People “Like”: Analysis of Social Media Strategies Used by Food Industry Brands, Lifestyle Brands, and Health Promotion Organizations on Facebook and Instagram
Source: J Med Internet Res. 2018 Jun 14;20(6):e10227. doi: 10.2196/10227 (PMC6024098; doi:10.2196/10227)
Supplement: Multimedia Appendix 3 [file jmir_v20i6e10227_app3.pdf]

Multimedia Appendix 3. Coding framework characteristics or strategies used for social media posts.

Strategy category	Specific strategy used	Health promotion organizations	Food industry brands		Lifestyle brands		Total	*P*[a]
		Facebook (n=34)	Facebook (n=51)	Instagram (n=28)	Facebook (n=58)	Instagram (n=56)	Both channels (n=227)	
**Format,n, (%)**								.612
	Photo	21 (62)	27 (53)	25 (89)	29 (50)	43 (77)	145 (64)	
	Video	11 (32)	23 (45)	3 (11)	27 (47)	13 (23)	77 (34)	
	Text	2 (6)	1 (2)	0 (0)	2 (3)	0 (0)	5 (2)	
**Content Topic, n (%)**								<.001
	Food/beverage	26 (76)	48 (94)	27 (96)	22 (38)	21 (38)	144 (63)	
	Body image	0 (0)	0 (0)	0 (0)	6 (10)	12 (21)	18 (8)	
	Physical activity	7 (21)	0 (0)	0 (0)	8 (14)	9 (16)	24 (11)	
	Weight loss	0 (0)	0 (0)	0 (0)	6 (10)	2 (4)	8 (4)	
	Other	1 (3)	3 (6)	1 (4)	16 (28)	12 (21)	33 (15)	
**Prompting engagement, n (%)**								.001
	Question	12 (35)	17 (33)	6 (21)	8 (14)	8 (14)	51 (22)	
	Action	10 (29)	7 (14)	3 (11)	12 (21)	4 (7)	36 (16)	
**Relationship Building n, (%)**								.212

	Building a relationship with users/fans/followers	5 (15)	3 (6)	6 (21)	8 (14)	0 (0)	22 (10)	
	Relatable	9 (26)	9 (18)	3 (11)	14 (24)	18 (32)	53 (23)	
**Emotion-Inducing, n, (%)**								<.001
	Appetite-inducing	6 (18)	43 (84)	21 (75)	14 (24)	21 (38)	105 (46)	
	Positive	14 (41)	7 (14)	5 (18)	37 (64)	31 (55)	94 (41)	
	Negative	4 (12)	0 (0)	0 (0)	0 (0)	1 (2)	5 (2)	
**Real-world tie-ins, n (%)**								<.001
	Yes	19 (56)	5 (10)	7 (25)	8 (14)	15 (27)	54 (24)	
**Encouragement to eat, n (%)**								<.001
	Recipe given	2 (6)	4 (8)	0 (0)	11 (19)	11 (20)	28 (12)	
	Food shown	7 (21)	22 (43)	23 (82)	7 (12)	11 (20)	70 (31)	
	Encouragement to make balanced choices or drink water	12 (35)	0 (0)	0 (0)	1 (2)	0 (0)	13 (6)	
	Occasion-specific	3 (9)	17 (33)	3 (11)	0 (0)	0 (0)	23 (10)	
**Encouragement to exercise, n (%)**								<.001
	Encourage exercise	8 (24)	0 (0)	0 (0)	14 (24)	10 (18)	32 (14)	
**Link to purchasable or downloadable item(s), n (%)**								<.001
	Purchasable link	1 (3)	49 (96)	1 (4)	32 (55)	33 (59)	116 (51)	
**Link to health information, n (%)**								<.001

	Health link	31 (91)	0 (0)	0 (0)	10 (17)	12 (21)	53 (23)	
**Tone of post, n (%)**								<.001
	More serious	22 (65)	3 (6)	0 (0)	17 (29)	12 (21)	51 (22)	
	Optimistic	12 (35)	48 (94)	28 (100)	41 (71)	44 (79)	173 (76)	
**Product promotion, n (%)**								<.001
		0 (0)	40 (78)	18 (64)	5 (9)	5 (9)	68 (30)	
**Strategies used, n (%)**								<.001
	Pop culture	1 (3)	7 (14)	2 (7)	10 (17)	1 (2)	21 (9)	
	Statistics/facts	12(35)	0 (0)	0 (0)	0(0)	0 (0)	12 (5)	
	Story	3 (9)	1 (2)	0 (0)	18 (31)	22 (39)	44 (19)	
	Visually appealing	3 (9)	18 (35)	15 (54)	13 (22)	25 (45)	74 (33)	
	Other	15 (44)	25 (49)	11 (39)	17 (29)	8 (14)	76 (33)	
**Diet assessment of food (ADG[b] food groups), n (%)**								<.001
	Discretionary foods	0 (0)	34 (67)	34 (67)	13 (22)	12 (21)	93 (69)	
	Dairy or protein-rich foods	0 (0)	0 (0)	0 (0)	4 (7)	5 (9)	9 (7)	
	Fruit and vegetables	9 (26)	0 (0)	0 (0)	1 (2)	3 (5)	13 (10)	
	Grains	3 (9)	8 (16)	8 (16)	1 (2)	0 (0)	20 (15)	
**Featuring people, n (%)**								<.001
	Featuring people	18 (53)	5 (10)	1 (4)	33 (57)	34 (61)	81 (36)	
**Promoted Post Detection, n (%)**								<.001

	Organic	34 (100)	25 (49)	28 (100)	50 (86)	56 (100)	193 (85)	
	Paid	0 (0)	26 (51)	0 (0)	8 (14)	0 (0)	34 (15)	
**Hashtag(s) used, n (%)**		26 (76)	13 (25)	10 (36)	9 (16)	33 (59)	91 (40)	<.001
**Text word count (words), mean (SD)**		43.32 (19.74)	31.69 (26.99)	19.00 (14.25)	74.40 (182.07)	61.54 (91.37)	50.00 (105)	.026[c]
**Video duration (minutes), mean, SD**		1.06 (1.24)	0.37 (0.79)	0.13 (0.09)	1.61 (2.86)	0.55 (0.39)	0.92 (1.86)	.147[c]

[a]Chi-square test

[b]ADG: Australian Dietary Guidelines [28]

[c]One-way analysis of variance (ANOVA) test with post-hoc Bonferoni test, significant differences between food industry and lifestyle
